# Fast regulation of the NF-κB signalling pathway in human skeletal muscle revealed by high-intensity exercise and ischaemia at exhaustion: Role of oxygenation and metabolite accumulation

**DOI:** 10.1016/j.redox.2022.102398

**Published:** 2022-07-08

**Authors:** Angel Gallego-Selles, Victor Galvan-Alvarez, Miriam Martinez-Canton, Eduardo Garcia-Gonzalez, David Morales-Alamo, Alfredo Santana, Juan Jose Gonzalez-Henriquez, Cecilia Dorado, Jose A.L. Calbet, Marcos Martin-Rincon

**Affiliations:** a Department of Physical Education, University of Las Palmas de Gran Canaria, Campus Universitario de Tafira s/n, Las Palmas de Gran Canaria, 35017, Spain; b Research Institute of Biomedical and Health Sciences (IUIBS), University of Las Palmas de Gran Canaria, Canary Islands, Spain; c Complejo Hospitalario Universitario Insular-Materno Infantil de Las Palmas de Gran Canaria, Clinical Genetics Unit, 35016, Las Palmas de Gran Canaria, Spain; d Department of Mathematics, University of Las Palmas de Gran Canaria, Campus Universitario de Tafira s/n, Las Palmas de Gran Canaria, 35017, Spain; e Department of Physical Performance, Norwegian School of Sport Sciences, Oslo, Norway

**Keywords:** Fatigue, ROS, NFĸB, Performance, Free radicals, Hypoxia

## Abstract

The NF-κB signalling pathway plays a critical role in inflammation, immunity, cell proliferation, apoptosis, and muscle metabolism. NF-κB is activated by extracellular signals and intracellular changes in Ca^2+^, P_i_, H^+^, metabolites and reactive oxygen and nitrogen species (RONS). However, it remains unknown how NF-κB signalling is activated during exercise and how metabolite accumulation and PO_2_ influence this process. Eleven active men performed incremental exercise to exhaustion (IE) in normoxia and hypoxia (P_I_O_2_:73 mmHg). Immediately after IE, the circulation of one leg was instantaneously occluded (300 mmHg). Muscle biopsies from m. *vastus lateralis* were taken before (Pre), and 10s (Post, occluded leg) and 60s after exercise from the occluded (Oc1m) and free circulation (FC1m) legs simultaneously together with femoral vein blood samples. NF-κB signalling was activated by exercise to exhaustion, with similar responses in normoxia and acute hypoxia, as reflected by the increase of p105, p50, IKKα, IκBβ and glutathione reductase (GR) protein levels, and the activation of the main kinases implicated, particularly IKKα and CaMKII δ_D_, while IKKβ remained unchanged. Postexercise ischaemia maintained and stimulated further NF-κB signalling by impeding muscle reoxygenation. These changes were quickly reverted at the end of exercise when the muscles recovered with open circulation. Finally, we have shown that Thioredoxin 1 (Trx1) protein expression was reduced immediately after IE and after 1 min of occlusion while the protein expression levels of glutathione peroxidase 1 (Gpx1) and thioredoxin reductase 1 (TrxR1) remained unchanged. These novel data demonstrate that exercising to exhaustion activates NF-κB signalling in human skeletal muscle and regulates the expression levels of antioxidant enzymes in human skeletal muscle. The fast regulation of NF-κB at exercise cessation has implications for the interpretation of published studies and the design of new experiments.

## Highlights

• NF-κB plays a critical role in inflammation, immunity, cell proliferation, apoptosis and muscle metabolism.
• Exercise to exhaustion in normoxia and severe hypoxia activates NF-κB signalling similarly.
• This activation entails increases of p105, p50, IKKα, IκBβ, and glutathione reductase protein levels and CaMKII δ_D_ phosphorylation.
• These changes are reverted within 60 s from the end of the exercise unless immediate ischaemia is applied at exhaustion.
• The fast regulation of the NF-κB signalling with the cessation of exercise should be considered to interpret published studies.

## Graphical abstract

**Figure:**
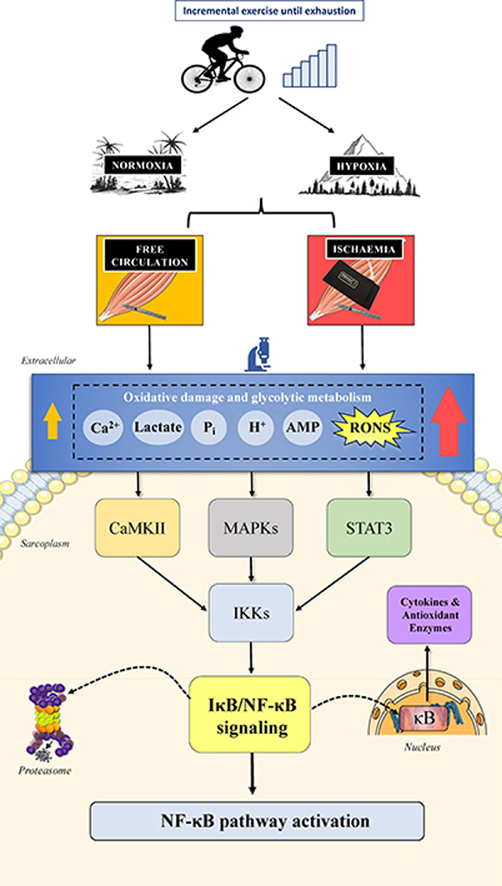
Schematic outline of the fast regulation of NF-κB signalling by exercise to exhaustion in normoxia and severe acute hypoxia in human skeletal muscle, revealed by ischaemia application at exhaustion.

## Abbreviations

Ca^2+^: calcium ion
CaMKII: calcium/calmodulin-dependent protein kinase II
ERK: extracellular-signal-regulated kinase
F_I_O_2_: inspired oxygen fraction
Gpx1: Glutathione peroxidase 1
GR: Glutathione reductase
H^+^: hydrogen ion
HRmax: maximal heart rate
Hyp: hypoxia
IE: incremental exercise to exhaustion
IKK: IκB kinase
IκB: inhibitor of nuclear factor κappa B
NF-κB: nuclear factor κappa-light-chain-enhancer of activated B cells
Nx: normoxia
p38 MAPK: p38 mitogen-activated protein kinases
p50: p50 subunit of NF-κB
p65: p65 (RelA) subunit of NF-κB
p105: p105 subunit of NF-κB
PCr: phosphocreatine
Pi: Inorganic Phosphorus
P_I_O_2_: partial pressure of inspired O_2_
RONS: reactive oxygen and nitrogen species
ROS: reactive oxygen species
Trx1: Thioredoxin 1
TrxR1: Thioredoxin Reductase 1
VO_2_: O_2_ consumption
VO_2_max: maximal O_2_ uptake
VO_2_peak: peak O_2_ uptake
Wmax: peak power output at exhaustion during the incremental exercise

## 1 Introduction

The transcription factor nuclear factor kappa-light-chain-enhancer of activated B cell (NF-κB) regulates over 150 genes involved in inflammation, immunity, cell proliferation, apoptosis [[1], [2], [3], [4]], and muscle metabolism [[4], [5], [6], [7]]. NF-κB is activated by extracellular signals, mostly cytokines, as well as intracellular changes in calcium [8] and reactive oxygen and nitrogen species (RONS) [[9], [10], [11]]. Although these signals are present in contracting muscles, contradicting findings have been reported regarding the effect of exercise on NF-κB activation and signalling. Exercise activates NF-κB in rodents, although this response is not homogeneous in all exercised muscles [12] and associated with muscle damage [13,14]. In humans, increased [15,16], unchanged [17] and decreased [18] NF-κB signalling has been reported after acute endurance [15,17] and resistance [16,18] exercise. Part of these discrepancies could be accounted for by differences in exercise protocol, characteristics of the subjects and collection timing of the muscle biopsies, which may influence metabolite accumulation and redox balance. It remains unknown whether a certain level of metabolite accumulation and RONS production is necessary to trigger an acute signalling response by NF-κB.

RONS production and metabolite build-up is exacerbated when the exercise is performed in hypoxia [19,20]. Cell culture experiments indicate that NF-κB [5,[21], [22], [23]] is stimulated by hypoxia and RONS. Nevertheless, whether metabolite accumulation and muscle oxygenation influence the NF-κB signalling response to exercise remains unknown. There is some experimental evidence in rodent muscle indicating that the exercise activation of NF-κB is produced through phosphorylation and activation of IKK by the RONS-sensitive upstream kinases ERK1/2 and p38 mitogen-activated protein kinase (p38 MAPK) [12,24]. However, data in humans are not conclusive [17]. In turn, NF-κB activation has been shown to induce the expression of some antioxidant enzymes, like Gpx1 and Trx1 [[25], [26], [27]], although information about this effect in human skeletal muscle *in vivo* is lacking. Glutathione reductase (GR) catalyses the reduction of the oxidized glutathione (GSSG) to reduced glutathione (GSH), and its activity is increased by oxidative stress in skeletal muscle [28] and by activation of Nrf2 signalling [29]. GR expression may be increased to facilitate the restoration of GSH during exercise conditions eliciting oxidative stress, and hence and increased expression of GR could be used as a biomarker of oxidative stress. Nevertheless, no previous study has determined the effects of intense exercise on the protein levels of GR in humans.

It has been reported that thioredoxin reductase 1 (TrxR1) may facilitate NF-κB signalling [30]. TrxR1 has been shown to be unchanged in human skeletal muscle after prolonged aerobic exercise [31] and increased after repeated sprint exercise ^32.^ Whether skeletal muscle TrxR1 expression increases during incremental exercise to exhaustion in normoxia and hypoxia remains unknown.

Therefore, the primary aim of this study was to determine whether NF-κB signalling is activated by acute exercise to exhaustion in human skeletal muscle and whether muscle oxygenation and metabolite accumulation play a role in this process. Another aim was to determine the time course of NF-κB signalling during the early recovery and ascertain whether NF-κB signalling remains activated by the application of post-exercise ischaemia. We hypothesized that NF-κB signalling is more markedly activated during exercise in severe acute hypoxia and further activated during post-exercise ischaemia and would be accompanied by upregulation of antioxidant enzymes regulated by NF-κB. We also hypothesized that NF-κB signalling would return to pre-exercise levels within 1 min of the termination of exercise when the muscles recover without occlusion of the circulation.

## 2 Methods

### 2.1 Subjects

Eleven young men volunteered to participate in this study (means ± SD; age: 21.5 ± 2.0 years, body mass: 72.3 ± 9.3 kg, height: 174 ± 8 cm, and body fat: 16.1 ± 4.9%). The inclusion criteria were: a) age between 18 and 35 years, b) sex: male, c) body mass index: < 30 kg m^−2^ all, d) normal 12-lead electrocardiogram, and e) having a physically active lifestyle exercising regularly 2–4 times a week, but without following a specific training program; and the exclusion criteria: a) smoking, b) any disease o allergy, c) any medical contraindication for exercise, d) being under any medical treatment [33]. All volunteers signed a written consent after receiving information about the aims and potential risk of the study. The study commenced after approval by the Ethical Committee of the University of Las Palmas de Gran Canaria and was carried out according to the Declaration of Helsinki. Subjects were asked to avoid ingesting caffeine and taurine-containing drinks, alcohol and exercise 24 h before the experiments. Besides, they recorded their dinner on the day before the first experimental session to repeat a similar diet on subsequent experimental sessions. Subjects were asked to maintain their usual diet until the end of the study.

### 2.2 Study design

Although this research was initially designed to determine the mechanisms that limit performance during whole-body exercise in humans previously published [[34], [35], [36], [37]], it was also planned to analyse the main signalling pathways activated by cellular stress during exercise and post-exercise ischaemia. In a recent paper, we focussed on Nrf2 mechanisms of activation/deactivation during exercise and recovery [33]. The present paper contains novel results regarding the mechanisms regulating NF-κB signalling during exercise in normoxia and severe acute hypoxia.

### 2.3 Pre-test and familiarization

Anthropometric and DEXA body composition assessments were performed (Hologic QDR-1500, software version 7.10, Hologic Corp., Waltham, MA, USA) [37] during the first visit to the laboratory, followed by familiarization with the exercise protocol. This was continued by two sessions to determine their maximal power at exhaustion (Wmax), the peak oxygen consumption (VO_2_peak), and maximal heart rate (HRmax) in normoxia (Nx; F_I_O_2_ = 0.21; P_I_O_2_ ∼143 mmHg) and hypoxia (Hyp; F_I_O_2_ = 0.104; P_I_O_2_ ∼73 mmHg) using a ramp incremental exercise test to exhaustion on a cycle ergometer (Lode Excalibur Sport 925900, Groningen, The Netherlands) [37]. VO_2_ was measured breath-by-breath with a metabolic cart (Vmax N29; Sensormedics, Yorba Linda, CA, USA) which was calibrated according to the manufacturer's instructions, using high-grade calibration gases (Carburos Metálicos, Las Palmas de Gran Canaria, Spain) [37]. The accuracy and precision of the metabolic cart was determined using a butane combustion test as previously described [38]. The highest 20s-averaged VO_2_ registered during the test was taken as the VO_2_peak [39].

### 2.4 Main experiments

**Fig. 1:**
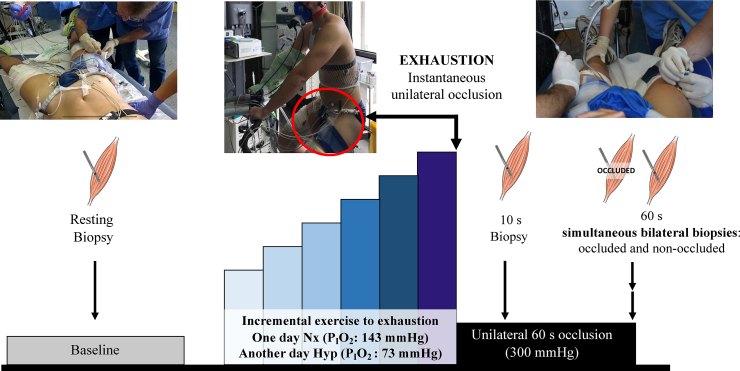
**Schematic illustration of the experimental protocol.** Eleven subjects performed an incremental exercise to exhaustion either in normoxia (Nx; F_I_O_2_ = 0.21) or in severe normobaric hypoxia (Hyp; F_I_O_2_ = 0.104) in random order. A resting skeletal muscle biopsy was obtained from the m. *vastus lateralis* before warm-up, followed by an incremental exercise test until exhaustion. Immediately at exhaustion, one leg was occluded at 300 mmHg and maintained during 60 s. Subsequent biopsies were taken from the occluded leg at 10 s and 60 s of occlusion in both trials (Nx and Hyp). In the test performed in hypoxia, the biopsies were taken bilaterally from the occluded leg and the leg recovering with free circulation 60 s after exercise cessation, while the subjects recovered breathing normoxic air.

Two main experimental sessions including one incremental exercise to exhaustion, one performed in normoxia (Nx; F_I_O_2_ = 0.21; barometric pressure 735–745 mmHg) and another in hypoxia (Hyp; F_I_O_2_ = 0.104; barometric pressure 735–745 mmHg) were carried out on separate days and random order (Fig. 1). During the tests, subjects were requested to maintain a pedalling rate close to 80 rpms. In both sessions, exhaustion (also task failure hereafter) was defined by the subject stopping pedalling suddenly or a pedalling rate below 50 rpm despite strong verbal encouragement for 5 s. The duration of the incremental exercise test to exhaustion was 15 ± 3 min in normoxia and 12 ± 4 min in hypoxia.

On the main experimental days, volunteers reported to the laboratory at 08.00 h, following an overnight fast. On the Nx day, a first basal muscle biopsy was obtained from the m. *vastus lateralis* of one of the two thigs, assigned randomly. This biopsy was labelled as Pre Nx. The needle was directed distally for the first biopsy, with 45° inclination [40]. Then a 5 mm incision was performed in the contralateral leg to obtain fast post-exercise muscle biopsies from both legs. Both incisions were covered with temporary plasters easy to remove at exhaustion. After that, a cuff (SCD10, Hokanson, Bellevue, WA, USA) connected to a rapid cuff inflator (Hokanson, E20 AG101) was placed around the thigh biopsied first and taped as close as possible to the inguinal crease. Then, the subjects moved to the cycle ergometer, and after verification of proper connections and readings from the instruments, and a 2-min data collection phase, the exercise test in normoxia was started at 80 W for 2 min and increased by 30 W every 2 min until task failure. At this moment, the cuff was inflated instantaneously at 300 mmHg, and a countdown started to obtain a second biopsy (labelled as Post Nx, second biopsy) exactly 10 s after exhaustion, i.e., after 10 s of complete ischaemia. For this second biopsy, the needle was introduced perpendicular to the thigh. Then, the subject rested quietly on the cycle ergometer while maintaining the cuff inflated, and exactly 60 s after the end of the exercise, the needle was introduced with 45° inclination towards the head to obtain the third biopsy (named as Oc1m Nx) [40]. This last biopsy allowed assessing muscle signalling changes during 60-s ischaemia, while metabolites from the anaerobic metabolism build-up and mitochondrial PO_2_ decreased to zero [37].

On the Hyp day, the first muscle biopsy was obtained while the subjects were breathing normoxic room air (Pre Hyp biopsy). The exercise test in hypoxia began with a 2-min recording period at rest (P_I_O_2_ ∼73 mmHg; AltiTrainer200, SMTEC, Nyon, Switzerland), followed by 2 min at 60 W, and increments of 20 W every 2 min until task failure. At this point, the cuff was instantaneously inflated, and the subjects switched to breath normoxic room air for the rest of the test. On the 10th s after the end of the exercise, the second biopsy was obtained (Post Hyp biopsy). Thereafter, the volunteers were moved to a stretcher while maintaining the cuff inflated to obtain the third muscle biopsy (Oc1m Hyp biopsy) exactly after 60 s of ischaemia. Simultaneously with the third, a fourth biopsy was taken from the contralateral thigh (FC1m), recovering with free circulation in normoxia during 60 s. This means that one leg recovered for 60 s in ischaemia and the other did so with an intact circulation. All biopsies were immediately frozen in liquid nitrogen and stored at −80 °C. We failed to obtain the biopsy corresponding to OC1M in two volunteers. In addition, due to scarce biopsy material, some assessments could not be done at all points for all subjects.

### 2.5 Muscle metabolites, protein extraction and Western blotting

Muscle metabolites and protein extracts were analysed as reported elsewhere [37], and total protein content was quantified using the bicinchoninic acid assay [41]. Briefly, ∼10 mg of muscle were ground by stainless steel balls during 1 min in a Mikro-Dismembrator S (Sartorius, Goettingen, Germany) and immediately homogenised in urea lysis buffer (6 M urea, 1% SDS) and 50X Complete protease inhibitor (Cat. #11697498001) and 10X PhosSTOP phosphatase inhibitor (Cat. #4906837001) cocktails (Roche, Basel, Switzerland). Almost equal final concentration in all muscle protein extracts was acquired by following an individual adjustment of the extract volume using a volume calibration curve. Then, the lysate was centrifuged for 12 min at 25,200 g at 16 °C. The resulting supernatant was diluted with electrophoresis loading buffer (160 mM Tris-HCl, pH 6.8, 5.9% SDS, 25.5% glycerol, 15% β-mercaptoethanol-bromophenol blue).

The optimal amount of total protein to be loaded and the antibody concentration for each assay was determined by loading protein from control and experimental samples in different amounts ranging from 2 to 35 μg. After verification of linearity within this range, equal amounts of protein of each sample (5–30 μg) were electrophoresed on SDS-PAGE gels using the system of Laemmli and transferred to Immun-Blot polyvinylidene fluoride (PVDF) membranes for protein blotting (Bio-Rad Laboratories, Hercules, CA, USA) (Supplementary Table 1). Control samples (whole skeletal muscle lysates from healthy young men) were prepared and run as the experimental samples. A total protein staining technique (Reactive Brown 10, Sigma Aldrich, St. Louis, MO, USA) was used to accurately quantify the variability of the assays and ensure optimal loading and transfer efficiency. For protein expression determination, the samples from each subject were run together onto the same gel intercalated with four control samples.

**Fig. 2:**
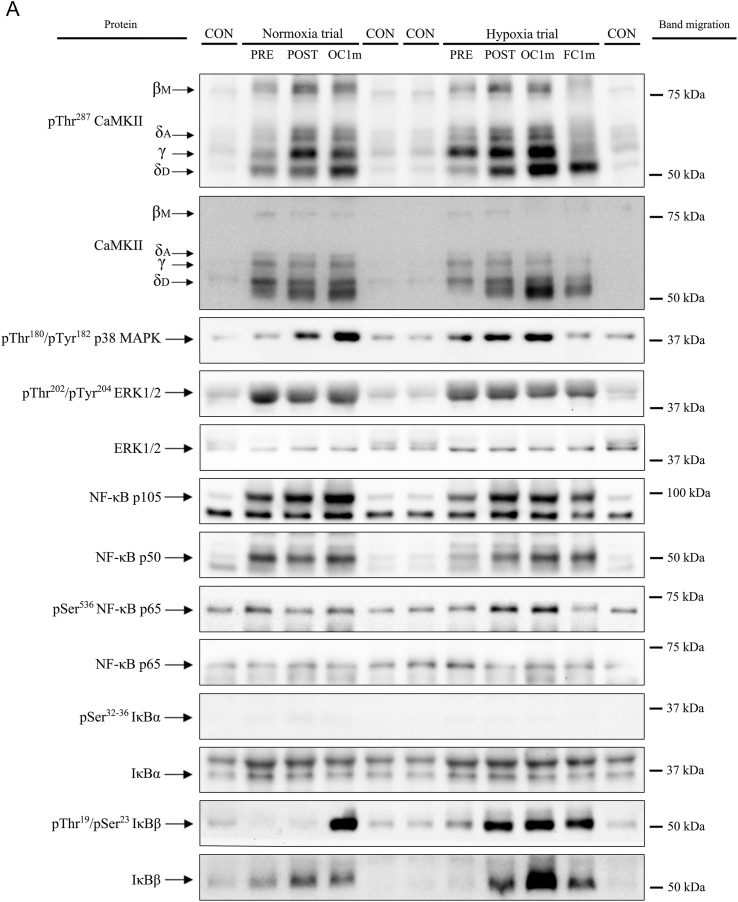

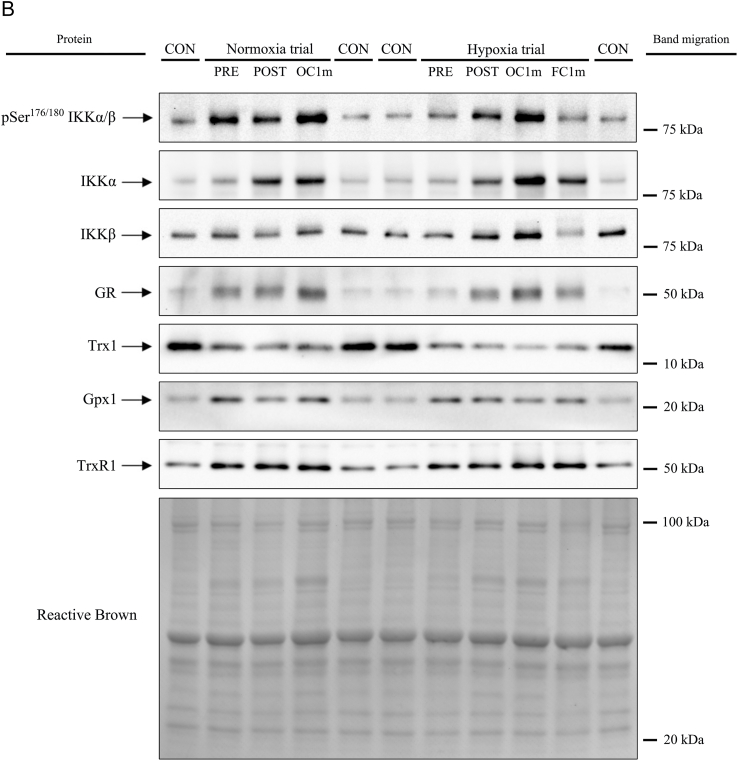
Representative images of protein expression levels (Western Blot) for all proteins studied, their regulatory phosphorylations and total amount of protein loaded (Reactive Brown Staining) for a single study participant. Images from top to bottom: pThr^287^ CaMKII, Total CaMKII, pThr^180^/Tyr^182^ p38 MAPK, pThr^202^/Tyr^204^ ERK1/2, NF-κB p105, NF-κB p50, NF-κB p65, pSer^536^ NF-κB p65, pSer^32/36^ IκBα, Total IκBα, pThr^19^/Ser^23^ IκBß, Total IκBß, pSer^176/180^ IKKα/β, Total IKKβ, Total IKKα, GR, Txr1, Gpx1, TrxR1 and Reactive Brown (as total protein loading control). Detailed description of experimental phases is included in Fig. 1. CON, non-intervention healthy human sample included in quadruplicate onto each gel as a loading control. Normoxia; test performed with F_I_O_2_ = 0.21, Hypoxia; test performed with F_I_O_2_ = 0.104; Pre, before exercise; Post, 10 s after the end of exercise with ischaemic recovery; Oc1m, 60 s after the end of exercise with ischaemic recovery; FC1m, 60 s after the end of exercise without ischaemic recovery (free circulation). The molecular weight standard markers closest to the migration of the band are indicated on the right side of the panel.

Membranes were blocked for 1 h in either 4% bovine serum albumin or 2.5–5% non-fat dried milk powder (blotting grade blocker) diluted in Tris-buffered saline containing 0.1% Tween 20 (TBS-T) (BSA-or Blotto-blocking buffer) and incubated overnight for 12–15 h at 4 °C with primary antibodies. Antibodies were diluted in 4% BSA-blocking buffer, 2.5 or 5% Blotto-blocking buffer. After incubation with primary antibodies, the membranes were incubated with an HRP-conjugated anti-rabbit or anti-mouse antibody (diluted 1:5000 to 1:20000 in 5% Blotto blocking buffer) and subsequent chemiluminescent visualization using Clarity™ Western ECL Substrate (Bio-Rad Laboratories, Hemel Hempstead, Hertfordshire, UK) using a ChemiDoc™ Touch Imaging System (Bio-Rad Laboratories, Hercules, CA, USA). Finally, band densitometric data were quantified in an exposition prior to saturation of the signal with the Image Lab © software 6.0.1 (Bio-Rad Laboratories, Hercules, CA, USA) as arbitrary units (a.u). Since loading was homogeneous in all membranes, no further corrections were performed. Representative immunoblots are depicted in Fig. 2.

### 2.6 Materials

The Protein Plus Precision All Blue Standards were acquired from Bio-Rad Laboratories (Hemel Hempstead Hertfordshire, UK). The antibodies employed in this investigation were obtained from different manufacturers. The corresponding catalogue numbers from Abcam (Cambridge, USA) were as follows:, IĸB beta total (no. ab109509) and Gpx1 (no. ab108429). The antibodies purchased from Cell Signalling Technology (Danvers, MA, USA) were: pThr^287^ CaMKII (no. 12716), Total CaMKII (no. 4436), pThr^180^/Tyr^182^ p38 MAPK (no. 9211), pThr^202^/Tyr^204^ ERK 1/2 (no. 9106), Total ERK 1/2 (no. 9102), NF-κB p105 and p50 (no. 13586), Total NF-κB p65 (no. 3034), pSer^536^ NF-κB p65 (no. 3033), pSer^32/36^ IκBα (no. 9246), Total IκBα (no. 9242), pThr [19]/Ser [23] IκBß (no. 4921), pSer^176/180^ IKKα/β (no. 2697), Total IKKα (no. 2682), Total IKKβ (no. 2370) and Trx1 (no. 2429). Other antibodies were purchased from Proteintech (Rosemont, USA): GR (no. 18257-1-AP) and TxrR1 (no. 11117-1-AP). The secondary HRP-conjugated goat anti-rabbit (no. 111-035-144) and the HRP-conjugated goat anti-mouse (no.115-035-003) antibodies were acquired from Jackson ImmunoResearch (West Grove, PA, USA). A CaMKII δ isoform-specific antibody (anti- CaMKII delta isoform no. A010-55AP; Badrilla) was employed to distinguish between the γ and δ isoforms, as previously described [42]. See Supplementary Table 1 for a more detailed description of antibodies and procedures.

### 2.7 Statistical analysis

The Gaussian distribution of variables was determined with the Shapiro–Wilks test, and when required, data were transformed logarithmically before further analysis. The main effects and interactions were assessed using a two-way 3 × 2 repeated-measures ANOVA with time (Pre, Post, and Oc1m) and F_I_O_2_ (Normoxia and hypoxia) as within-subject factors. Additionally, when no significant differences were observed between the post-exercise conditions, the average of the means of the two Pre conditions was compared with those of post-exercise conditions (Post normoxia, Oc1m normoxia, Post hypoxia and Oc1m). For this purpose, a contrast analysis in a two way within repeated measures analysis was performed using R (R Foundation for Statistical Computing, Vienna, Austria). The differences between the occluded and non-occluded leg were determined using a paired *t*-test. The Mauchly's test of sphericity was applied before the ANOVAs. In the case of violation of the sphericity assumption, the degrees of freedom were adjusted according to the Huynh and Feldt test. When significant main or interaction effects were detected, pairwise comparisons at specific time points were adjusted for multiple comparisons using the Holm-Bonferroni procedure. Linear relationships between variables were examined using a linear mixed model, and the Likelihood Ratio Test for the random effects (LRT) was computed and reported with the marginal and conditional r-squared values. Unless otherwise stated, results are reported as the mean ± standard deviation (SD). Statistical significance was set at p < 0.05. Statistical analyses were performed using IBM SPSS Statistics v.21 for Mac (SPSS Inc., Chicago, IL, USA) and jamovi v1.8.1. (Jamovi project, 2021).

## 3 Results

### 3.1 Muscle metabolites

During the incremental exercise to exhaustion, subjects reached 287.3 ± 39 and 177.3 ± 36.4 W in normoxia and hypoxia, respectively (p < 0.001). The effects of metabolite accumulation in both conditions have been reported previously [37]. Briefly, muscle lactate, phosphocreatine (PCr) and ATP changed similarly after IE. Muscle lactate increased only at Oc1m (25%; p < 0.05), and PCr was reduced by a 94 and 48% in Oc1m and FC1m, respectively (p < 0.005). Femoral vein PO_2_ was 21.1 ± 2.0 and 10.6 ± 2.8 mmHg at Wmax, in Nx and Hyp, respectively (p < 0.001).

### 3.2 Muscle signalling

#### 3.2.1 pThr^287^ CaMKII muscle isoforms

**Fig. 3:**
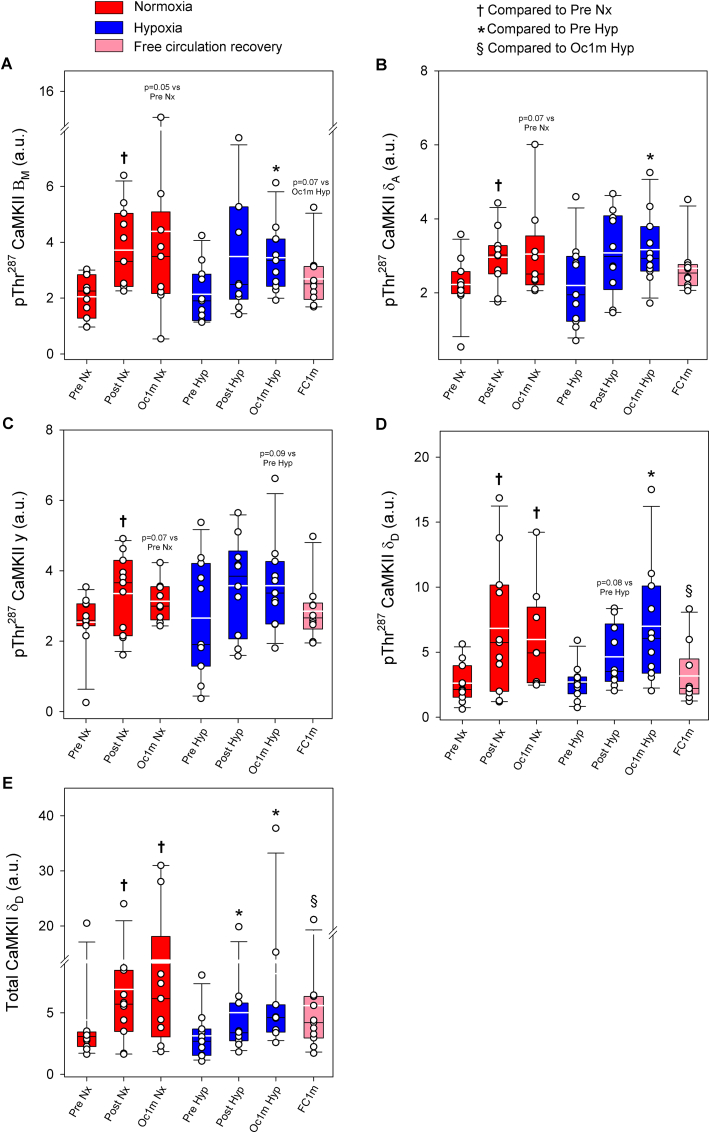
**CaMKII isoforms phosphorylation and total CaMKII δ**_**D**_**in human skeletal muscle in response to incremental exercise to exhaustion in normoxia and severe hypoxia, and post-exercise ischaemia.** Levels of protein expression of (A) pThr^287^ CaMKII β_M_, (B) pThr^287^ CaMKII γ, (C) pThr^287^ CaMKII δ_A_, (D) pThr^287^ CaMKII δ_D_ and (E) Total CaMKII δ_D_. Nx: normoxia session (F_I_O_2_ = 0.21, P_I_O_2_ = 143 mmHg); Hyp: severe normobaric hypoxia session (F_I_O_2_ = 0.104, P_I_O_2_ = 73 mmHg); Pre: before exercise; Post: 10 s after exercise cessation during ischaemic recovery; Oc1m: 60 s after exercise cessation during ischaemic recovery; FC1m: 60 s after exercise cessation during recovery with free circulation. n = 11 for all conditions except for Oc1m Nx (n = 9), Post Hyp (n = 10) and FC1m (n = 10). See Fig. 1 for a detailed description of the experimental phases. The statistical analysis was performed with logarithmically transformed data for pThr^287^ CaMKII β_M,_ pThr^287^ CaMKII δ_A_ and Total CaMKII δ_D_. Values presented are means ± standard errors and expressed in arbitrary units (a.u.). †p < 0.05 vs. Pre Nx; *p < 0.05 vs. Pre Hyp; §p < 0.05 vs. Oc1m Hyp.

Compared to Pre, pThr^287^ CaMKII β_Μ_ was increased by 1.6 and 2.0-fold after IE and 1 min of occlusion, respectively, with a similar response in Nx and Hyp (F_I_O_2_ effect p = 0.96, time effect p = 0.005, F_I_O_2_ x time interaction p = 0.92). pThr^287^ CaMKII β_Μ_ returned to pre-exercise values after 1-min recovery with open circulation (Fig. 3A). Compared to Pre, pThr^287^ CaMKII δ_A_ was increased by 1.3 and 1.5-fold after IE and 1 min of occlusion, respectively, with a similar response in Nx and Hyp (F_I_O_2_ effect p = 0.74, time effect p = 0.014, F_I_O_2_ x time interaction p = 0.93). pThr^287^ CaMKII δ_A_ returned towards pre-exercise values after 1-min recovery with open circulation (p = 0.24, compared to Pre levels) (Fig. 3B). Compared to Pre, pThr^287^ CaMKII γ was increased by 1.3 and 1.4- fold after IE and 1 min of occlusion, respectively (F_I_O_2_ effect p = 0.16, time effect p = 0.022, F_I_O_2_ x time interaction p = 0.72). One minute after IE, the level of pThr^287^ CaMKII γ was similarly elevated in both legs (p = 0.10) (Fig. 3C). Compared to Pre, pThr^287^ CaMKII δ_D_ was increased by 2.0 and 2.5-fold after IE and 1 min of occlusion, respectively, with a similar response in Nx and Hyp (F_I_O_2_ effect p = 0.75, time effect p < 0.001, F_I_O_2_ x time interaction p = 0.79). pThr^287^ CaMKII δ_D_ returned to pre-exercise values after recovery for 1 min with open circulation (p = 0.73 compared to Pre) (Fig. 3D).

**Supplementary Fig. 1:**
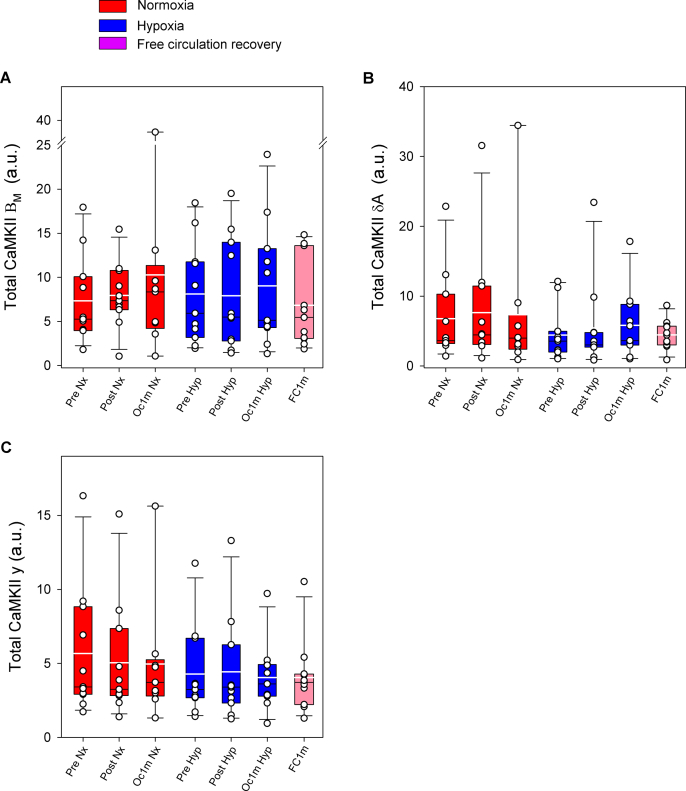
Total CaMKII isoforms in human skeletal muscle in response to incremental exercise to exhaustion in normoxia and severe hypoxia, and post-exercise ischaemia. Levels of protein expression of (A) Total CaMKII β_M_, (B) Total CaMKII δ_A,_ and (C) Total CaMKII γ. Total CaMKII δ_D_ is included in Fig. 2E. Nx: normoxia session (F_I_O_2_ = 0.21, P_I_O_2_ = 143 mmHg); Hyp: severe normobaric hypoxia session (F_I_O_2_ = 0.104, P_I_O_2_ = 73 mmHg); Pre: before exercise; Post: 10 s after exercise cessation during ischaemic recovery; Oc1m: 60 s after exercise cessation during ischaemic recovery; FC1m: 60 s after exercise cessation during recovery with free circulation. n = 11 for all conditions except for Oc1m Nx (n = 9). See Fig. 1 for a detailed description of the experimental phases. Values presented are means ± standard errors and expressed in arbitrary units (a.u.). No statistically significant effects were detected.

Total CaMKII δ_D_ was increased by 1.6 and 2.4-fold after IE and 1 min of occlusion, respectively (time effect p < 0.001), with a similar response in Nx and Hyp (F_I_O_2_ effect p = 0.09, F_I_O_2_ x time interaction p = 0.62) (Fig. 3E). Total CaMKII δ_D_ returned to pre-exercise values after 1-min recovery with open circulation (p = 0.13 compared to Pre). One min after exercise, total CaMKII δ_D_ was 46% lower in the leg with free circulation compared with the occluded leg (p = 0.014) (Fig. 3E). No significant changes were observed in the total expression of the other CaMKII isoforms (Supplementary Fig. 1).

### 3.3 p38 MAPK, ERK1/2 and NF-ĸB signalling

**Fig. 4:**
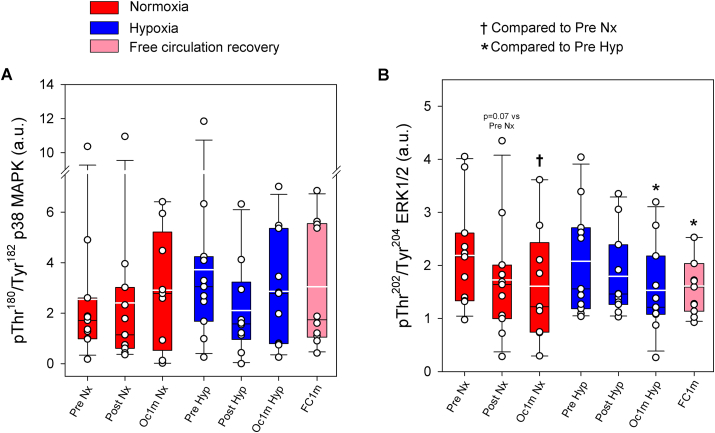
**p38 MAPK and ERK1/2 phosphorylation in human skeletal muscle in response to incremental exercise to exhaustion in normoxia and severe hypoxia, and post-exercise ischaemia.** Levels of protein expression of (A) pThr^180^/Tyr^182^ p38 MAPK and (B) pThr^202^/Tyr^204^ ERK1/2. Nx: normoxia session (F_I_O_2_ = 0.21, P_I_O_2_ = 143 mmHg); Hyp: severe normobaric hypoxia session (F_I_O_2_ = 0.104, P_I_O_2_ = 73 mmHg); Pre: before exercise; Post: 10 s after exercise cessation during ischaemic recovery; Oc1m: 60 s after exercise cessation during ischaemic recovery; FC1m: 60 s after exercise cessation during recovery with free circulation. For panel (A), n = 11 for all conditions except for Oc1m Nx (n = 9), Post Hyp (n = 10) and FC1m (n = 10) and for panel (B), n = 11 for all conditions except for Oc1m Nx (n = 9). See Fig. 1 for a detailed description of the experimental phases. The statistical analysis was performed with logarithmically transformed data for pThr^180^/Tyr^182^ p38 MAPK. Values presented are means ± standard errors and expressed in arbitrary units (a.u.). †p < 0.05 vs. Pre Nx; *p < 0.05 vs. Pre Hyp; #p < 0.05 vs. Post Hyp; §p < 0.05 vs. Oc1m Hyp.

No significant changes were observed in p38 MAPK phosphorylation at Thr^180^/Tyr^182^ (F_I_O_2_ effect p = 0.95, time effect p = 0.54, F_I_O_2_ x time interaction p = 0.25) (Fig. 4A). Compared to Pre, phospho-Thr^202^/Tyr^204^ ERK1/2 was reduced 17 and 24% after IE, and after 1 min of occlusion (time effect p = 0.007), with a similar response in normoxia and hypoxia (F_I_O_2_ effect p = 0.92, F_I_O_2_ x time interaction p = 0.62). After 1 min of recovery, phospho-Thr^202^/Tyr^204^ ERK1/2 was similar in both legs, regardless of the recovery with open or occluded circulation (p = 0.71) (Fig. 4B).

**Fig. 5:**
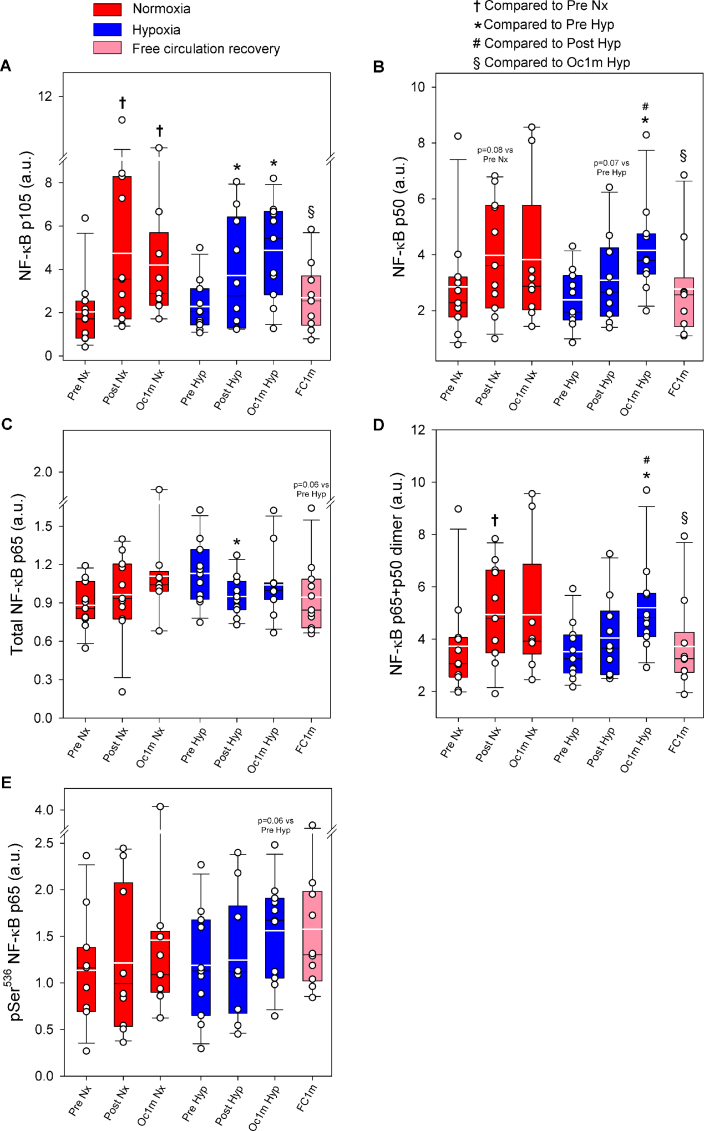
**NF-**κ**B signalling in human skeletal muscle in response to incremental exercise to exhaustion in normoxia and severe hypoxia, and post-exercise ischaemia.** Levels of protein expression of (A) NF-κB p105, (B) NF-κB p50, (C) Total NF-κB p65, (D) NF-κB p65 + p50 dimer and (E) pSer^536^ NF-κB. Nx: normoxia session (F_I_O_2_ = 0.21, P_I_O_2_ = 143 mmHg); Hyp: severe normobaric hypoxia session (F_I_O_2_ = 0.104, P_I_O_2_ = 73 mmHg); Pre: before exercise; Post: 10 s after exercise cessation during ischaemic recovery; Oc1m: 60 s after exercise cessation during ischaemic recovery; FC1m: 60 s after exercise cessation during recovery with free circulation. For panels (A), (B), (D) n = 11 for all conditions except for Oc1m Nx (n = 9), Post Hyp (n = 10) and FC1m (n = 10), for panel (C) n = 11 for all conditions except for Oc1m Nx (n = 9) and for panel (E) n = 11 for all conditions except for Post Nx (n = 10), Oc1m Nx (n = 9), Post Hyp (n = 10) and FC1m (n = 10), See Fig. 1 for a detailed description of the experimental phases. The statistical analysis was performed with logarithmically transformed data for NF-κB p105, Total NF-Κb p65 and NF-κB p65 + p50 dimer. Values presented are means ± standard errors and expressed in arbitrary units (a.u.). †p < 0.05 vs. Pre Nx; *p < 0.05 vs. Pre Hyp; #p < 0.05 vs. Post Hyp; §p < 0.05 vs. Oc1m Hyp.

Compared to Pre, p105 was increased by 1.9 and 2.1-fold after IE, and after 1 min of occlusion (time effect p < 0.001), with a similar response in normoxia and hypoxia (F_I_O_2_ effect p = 0.91, F_I_O_2_ x time interaction p = 0.58) (Fig. 5A). p105 returned to pre-exercise values after 1 min of recovery with open circulation (p = 0.44, Fig. 5A). Consequently, 1 min after exercise p105 was 47% lower in the leg with free circulation compared with the occluded leg (p = 0.011). p50 followed a similar pattern, increasing by 1.3 and 1.5-fold after IE, and after 1-min occlusion (time effect p = 0.005), respectively, with a similar response in normoxia and hypoxia (F_I_O_2_ effect p = 0.72, F_I_O_2_ x time interaction p = 0.32) (Fig. 5B). p50 returned to pre-exercise values after 1 min of recovery with open circulation (p = 0.45, Fig. 5B). Compared to the occluded leg, p50 was 33% lower in the leg recovering with free circulation (p = 0.003).

The total amount of p65 was unchanged immediately after IE, increasing by 11% during ischaemia, compared to the immediate post-exercise value (p = 0.014); time effect p = 0.032), with a similar response in normoxia and hypoxia (F_I_O_2_ effect p = 0.52, F_I_O_2_ x time interaction p = 0.14) (Fig. 5C). p65 returned to pre-exercise values after 1-min recovery with open circulation (p = 0.057, Fig. 5C). One min after exercise, p65 was similar in the leg recovering with free circulation and the ischaemic leg (p = 0.087).

The p65 + p50 was increased 1.2 and 1.4-fold after IE, and after 1 min of occlusion (time effect p = 0.006), respectively, with a similar response in normoxia and hypoxia (F_I_O_2_ effect p = 0.73, F_I_O_2_ x time interaction p = 0.37) (Fig. 5D). p65 + p50 returned to pre-exercise values after 1-min recovery with open circulation (p = 0.80, Fig. 5D). Compared to the occluded leg, p65 + p50 was 29% lower in the leg recovering with free circulation (p < 0.001). During the 1 min of ischaemia, p65 + p50 was increased by 17% (p = 0.046).

Phospho-Ser^536^ p65 was unchanged immediately after IE, and was increased 1.6 fold in the leg recovering with ischaemia, compared to the immediate post-exercise value (p = 0.023); time effect p = 0.006), with a similar response in normoxia and hypoxia (F_I_O_2_ effect p = 0.97, F_I_O_2_ x time interaction p = 0.55) (Fig. 5E). One min after exercise, Phospho-Ser^536^ p65 was similar in the leg recovering with free circulation and the ischaemic leg (p = 0.19) (Fig. 5E).

**Fig. 6:**
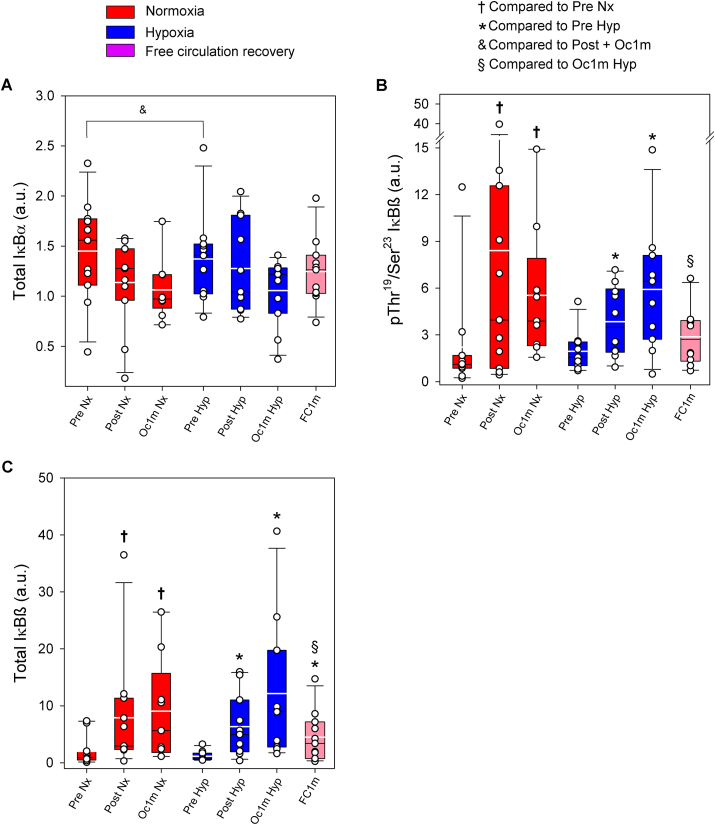
**IκB signalling in human skeletal muscle in response to incremental exercise to exhaustion in normoxia and severe hypoxia, and post-exercise ischaemia.** Levels of protein expression of (A) Total IκBα, (B) pThr^19^/Ser^23^ IκBß, and (C) Total IκBß. Nx: normoxia session (F_I_O_2_ = 0.21, P_I_O_2_ = 143 mmHg); Hyp: severe normobaric hypoxia session (F_I_O_2_ = 0.104, P_I_O_2_ = 73 mmHg); Pre: before exercise; Post: 10 s after exercise cessation during ischaemic recovery; Oc1m: 60 s after exercise cessation during ischaemic recovery; FC1m: 60 s after exercise cessation during recovery with free circulation. For panels (A) and (C), n = 11 for all conditions except for Oc1m Nx (n = 9) and for panel (B), n = 11 for all conditions except for Oc1m Nx (n = 9), Post Hyp (n = 10) and FC1m (n = 10). See Fig. 1 for a detailed description of the experimental phases. The statistical analysis was performed with logarithmically transformed data for Total IκBß. Values presented are means ± standard errors and expressed in arbitrary units (a.u.). & mean of the pre-exercise conditions in normoxia and hypoxia compared with the mean of the four post-exercise conditions (p = 0.008); †p < 0.05 vs. Pre Nx; *p < 0.05 vs. Pre Hyp; #p < 0.05 vs. Post Hyp; §p < 0.05 vs. Oc1m Hyp.

No significant changes were observed in the total amount of IĸBα protein (F_I_O_2_ effect p = 0.83, time effect p = 0.07, and F_I_O_2_ x time interaction p = 0.47) (Fig. 6A), while its phosphorylation remained below the detection levels in all conditions. However the average of the post-exercise conditions was 20% lower than the average of the two Pre conditions (p = 0.008). During the 1-min ischaemia the total amount of IĸBα protein was reduced by 13% when compared to Post (p = 0.019, *t*-test) (Fig. 6A).

Compared to Pre, IĸBβ Thr^19^/Ser^23^ phosphorylation was increased by 2.8 and 3.1-fold immediately after IE and after 1 min of occlusion, respectively, (time effect p = 0.01) (Fig. 6B). The response was similar in normoxia and hypoxia (F_I_O_2_ effect p = 0.78, F_I_O_2_ x time interaction p = 0.73). IĸBβ Thr^19^/Ser^23^ phosphorylation returned to pre-exercise values after 1 min of recovery with free circulation (p = 0.26, Fig. 6B). Compared to the occluded leg, IĸBβ Thr^19^/Ser^23^ phosphorylation was 54% lower in the leg recovering with free circulation (p = 0.026). The total amount of IĸBβ protein was increased by 4.5 and 5.8-fold immediately after IE and after 1 min of occlusion, respectively, (time effect p < 0.001) (Fig. 6C). The response was similar in normoxia and hypoxia (F_I_O_2_ effect p = 0.78, F_I_O_2_ x time interaction p = 0.68). Compared to the leg recovering in ischaemia, IĸBβ total protein was slightly reduced by 63% in the leg recovering with a free circulation (p = 0.015, Fig. 6C). However, 1 min after the end of exercise, the total amount of IĸBβ protein was 3.9-fold higher than pre-exercise values in the leg recovering with free circulation (p = 0.005).

**Fig. 7:**
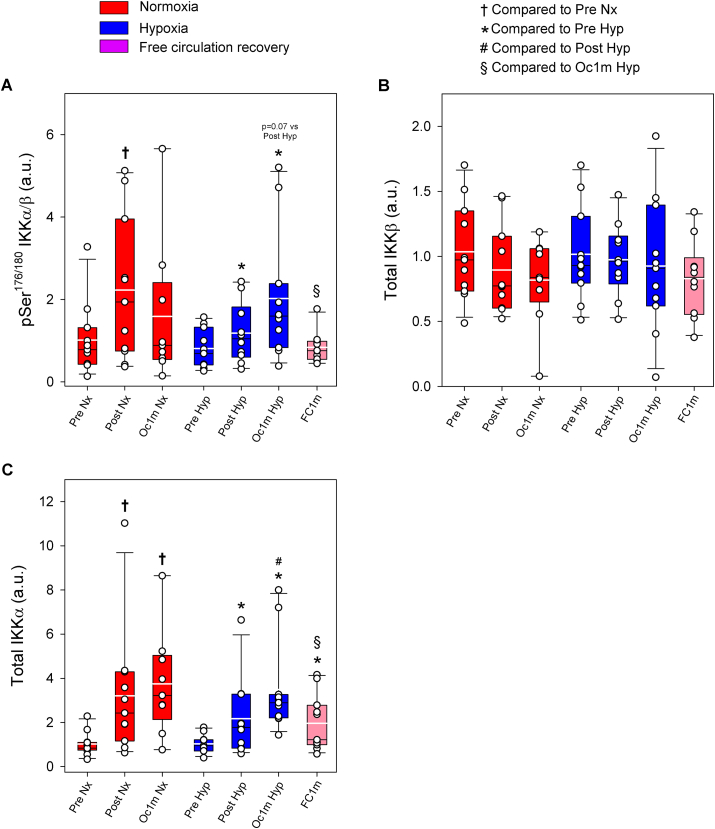
**IKK signalling in human skeletal muscle in response to incremental exercise to exhaustion in normoxia and severe hypoxia, and post-exercise ischaemia.** Levels of protein expression of (A) pSer^176/180^ IKKα/ß, (B) Total IKKβ, and (C) Total IKKα. Nx: normoxia session (F_I_O_2_ = 0.21, P_I_O_2_ = 143 mmHg); Hyp: severe normobaric hypoxia session (F_I_O_2_ = 0.104, P_I_O_2_ = 73 mmHg); Pre: before exercise; Post: 10 s after exercise cessation during ischaemic recovery; Oc1m: 60 s after exercise cessation during ischaemic recovery; FC1m: 60 s after exercise cessation during recovery with free circulation. For panels (A) and (B), n = 11 for all conditions except for Oc1m Nx (n = 9), Post Hyp (n = 10) and FC1m (n = 10) and for panel (C), n = 11 for all conditions except for Oc1m Nx (n = 9). See Fig. 1 for a detailed description of the experimental phases. The statistical analysis was performed with logarithmically transformed data for Total IKKα. Values presented are means ± standard errors and expressed in arbitrary units (a.u.). †p < 0.05 vs. Pre Nx; *p < 0.05 vs. Pre Hyp; #p < 0.05 vs. Post Hyp; §p < 0.05 vs. Oc1m Hyp.

Compared to Pre, IKKα/β Ser^176/180^ phosphorylation was increased by 1.8 and 2.0-fold immediately after IE and after 1 min of occlusion, respectively, (time effect p = 0.04) (Fig. 7A). The response was similar in normoxia and hypoxia (F_I_O_2_ effect p = 0.67, F_I_O_2_ x time interaction p = 0.34). IKKα/β Ser^176/180^ phosphorylation returned to pre-exercise values after 1 min of recovery with a free circulation (p = 0.87, Fig. 7A). Compared to the occluded leg, IKKα/β Ser^176/180^ phosphorylation was 61% lower in the leg recovering with free circulation (p = 0.019, Fig. 7A). IKKβ total protein did not change significantly (F_I_O_2_ effect p = 0.67, time effect p = 0.34, and F_I_O_2_ x time interaction p = 0.81) (Fig. 7B). Compared to Pre, IKKα total protein was increased by 2.6 and 3.5-fold immediately after IE and after 1 min of occlusion, respectively, (time effect p < 0.001) (Fig. 7C). The response was similar in normoxia and hypoxia (F_I_O_2_ effect p = 0.24, F_I_O_2_ x time interaction p = 0.40). IKKα total protein returned to pre-exercise values after 1 min of recovery with free circulation remaining 1.9-fold above Pre (p = 0.008, Fig. 7C). Compared to the occluded leg, IKKα total protein was 44% lower in the leg recovering with free circulation (p = 0.03).

### 3.4 Antioxidant enzymes

**Fig. 8:**
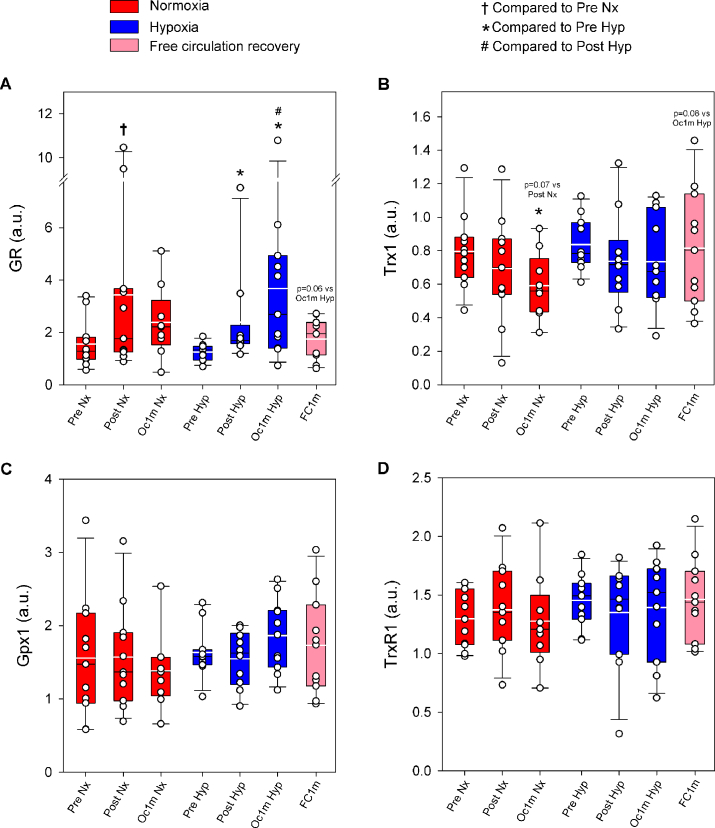
**Antioxidant enzymes in human skeletal muscle in response to incremental exercise to exhaustion in normoxia and severe hypoxia, and post-exercise ischaemia.** Levels of protein expression of (A) GR, (B) Trx1, (C) Gpx1 and (D) TrxR1. Nx: normoxia session (F_I_O_2_ = 0.21, P_I_O_2_ = 143 mmHg); Hyp: severe normobaric hypoxia session (F_I_O_2_ = 0.104, P_I_O_2_ = 73 mmHg); Pre: before exercise; Post: 10 s after exercise cessation during ischaemic recovery; Oc1m: 60 s after exercise cessation during ischaemic recovery; FC1m: 60 s after exercise cessation during recovery with free circulation. For panel (A), n = 11 for all conditions except for Oc1m Nx (n = 9) and Post Hyp (n = 10) and for panels (B), (C) and (D), n = 11 except for Oc1m Nx (n = 9). See Fig. 1 for a detailed description of the experimental phases. The statistical analysis was performed with logarithmically transformed data for GR. Values presented are means ± standard errors and expressed in arbitrary units (a.u.). †p < 0.05 vs. Pre Nx; *p < 0.05 vs. Pre Hyp; #p < 0.05 vs. Post Hyp.

Compared to Pre, glutathione reductase (GR) protein expression was increased by 2.1 and 2.2-fold immediately after IE and after 1 min of occlusion, respectively, (time effect p = 0.002) (Fig. 8A). The response was similar in normoxia and hypoxia (F_I_O_2_ effect p = 0.46, F_I_O_2_ x time interaction p = 0.53). GR protein expression returned to pre-exercise values after 1 min of recovery with a free circulation (p = 0.15, Fig. 8A). Compared to the occluded leg, GR protein expression was 52% lower in the leg recovering with free circulation (p = 0.059, Fig. 8A).

Compared to Pre, thioredoxin 1 (Trx1) protein expression was reduced by 10 and 17% immediately after IE and after 1 min of occlusion, respectively, (time effect p = 0.012) (Fig. 8B). The response was similar in normoxia and hypoxia (F_I_O_2_ effect p = 0.51, F_I_O_2_ x time interaction p = 0.77). After 1 min of recovery, Trx1 protein expression was similar in the legs recovering with ischaemia and free circulation (p = 0.083, Fig. 8B).

No significant changes were observed in protein expression levels of glutathione peroxidase 1 (Gpx1) (F_I_O_2_ effect p = 0.11, time effect p = 0.52; F_I_O_2_ x time interaction p = 0.50) (Fig. 8C). No significant changes were observed in protein expression levels of thioredoxin reductase 1 (TrxR1) (F_I_O_2_ effect p = 0.27, time effect p = 0.99; F_I_O_2_ x time interaction p = 0.48) (Fig. 8D).

### 3.5 Linear associations

Positive linear associations were observed between pThr^287^ CaMKII δ_D_ and pSer^176/180^ IKKα/β (R^2^ marginal = 0.52, R^2^ conditional = 0.74, intercept and slope random effect LRT p < 0.001), Total IKKα (R^2^ marginal = 0.58, R^2^ conditional = 0.92, intercept and slope random effect LRT p < 0.001), NF-κB p105 (R^2^ marginal = 0.55, R^2^ conditional = 0.83, intercept and slope random effect LRT p = 0.001), Total IĸB β (R^2^ marginal = 0.46, R^2^ conditional = 0.79, intercept and slope random effect LRT p < 0.001), Phospho-Ser^536^ p65 (R^2^ marginal = 0.07, R^2^ conditional = 0.46, intercept and slope random effect LRT P < 0.001) p50 + p65 (R^2^ marginal = 0.40, R^2^ conditional = 0.84, intercept and slope random effect LRT p = 0.004) and GR (R^2^ marginal = 0.42, R^2^ conditional = 0.90, intercept and slope random effect LRT p < 0.001). (Supplementary Figs. 2A, B, C, D, E, F and G).

A positive linear association was observed between p50 and its precursor p105 (R^2^ marginal = 0.45, R^2^ conditional = 0.85, intercept and slope random effect LRT p = 0.04), while pSer^176/180^ IKKα/β was linearly associated with Total IĸBβ with GR (R^2^ marginal = 0.30, R^2^ conditional = 0.74, intercept and slope random effect LRT p < 0.001) (Supplementary Figs. 3A and B).

## 4 Discussion

**Fig. 9:**
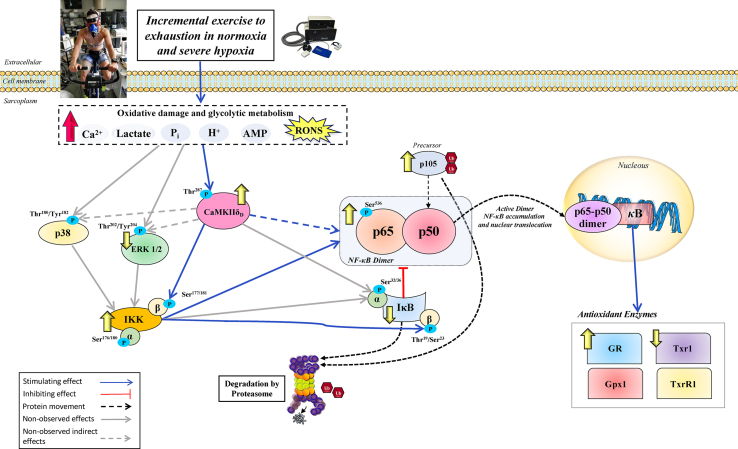
**Schematic representation of the measured mechanisms regulating NF-κB signalling in human skeletal muscle in response to exhaustive exercise in normoxia and severe hypoxia.** Extracellular and intracellular signals such as Ca^2+,^ lactate, H^+^, Pi, AMP and RONS evoked by an incremental exercise to exhaustion largely activated CaMKII. CaMKII activation reduces the inhibitory action of IκB proteins via phosphorylation, which targets them for proteasomal degradation by a direct or indirect mechanism (through CaMKIIδ_D_-mediated activation of IKKβ). This was accompanied by an increase in the total levels of IKKα, which should favour the nuclear translocation of the p65-p50 heterodimer and transcriptional regulation of NF-κB-responsive genes. The phosphorylation levels of the RONS-sensitive upstream kinases ERK1/2 and p38 MAPK were not elevated by the exhausting exercise test. Overall, the activation in NF-κB signalling was associated with increased and decreased GR and Trx1protein content, respectively. No changes were found for Gpx1 and TrxR1 after incremental exercise. Several key markers increased by exercise were rapidly downregulated within 60 s when the leg recovered with free circulation, demonstrating a fast regulation of NF-κB at exercise cessation which depends on muscle reoxygenation. None of the proteins studied were differentially modulated by performing exercise either in normoxia or severe hypoxia. Stimulating/inhibiting effects are represented by blue/red connecting lines (dashed if the effect is indirect). Known actions not observed in the present investigation are shown in grey (dashed if the effect is indirect). Changes in cellular locations are depicted with black dashed lines. The arrows in yellow shown beside the specific markers, illustrate the magnitude of the overall protein expression changes (increase/decrease) in this investigation. (For interpretation of the references to colour in this figure legend, the reader is referred to the Web version of this article).

This study shows that during incremental exercise to exhaustion, NF-κB signalling is activated to a similar extent in normoxia and severe acute hypoxia in human skeletal muscle. Importantly, NF-κB signalling remains stimulated during post-exercise ischaemia. However, most components of the NF-κB signalling pathway return to pre-exercise levels within 1 min after the finalization of the exercise when the muscles recover with a free circulation, demonstrating the O_2_-dependency of this process. These responses are closely associated with the activating phosphorylation of CaMKII δ_D_ and involve an increase of the protein expression of IKKα, IĸBβ, and glutathione reductase in skeletal muscle (Fig. 9). These findings highlight the importance of obtaining the muscle biopsies as close as possible to exhaustion and the usefulness of applying immediate post-exercise ischaemia to impede the recovery of this signalling cascade with the cessation of muscle contractile activity.

### 4.1 NF-κB signalling is activated during exercise to exhaustion in human skeletal muscle

NF-κB proteins consist of five members, including p65 protein (or RelA), RelB, c-Rel, p50 protein (or mature NF-κB1), and p52 protein (or mature NF-κB2), which form dimeric complexes that transactivate several target genes via binding to the κB enhancer [2,11]. NF-κB may be activated through the canonical and noncanonical pathways [43]. Canonical NF-κB activity depends on the heterodimer p65-p50 that consists of the transcriptional activator (p65 protein) and the protein p50, which is produced by constitutive proteasomal processing of the precursor p105 (or NF-κB1 precursor protein) [44]. The present investigation shows that the protein levels of p50 and its precursor p105 are elevated in human skeletal muscle by intense exercise indicating that exercise promotes upregulation of the transcription and translation of p105 and its subsequent proteasomal processing to produce p50. Interestingly, p50 and p105 increases with exercise were similar when the exercise was performed in normoxia and a simulated altitude of 5300 m above sea level. In contrast with our results, no changes in p50 and IκBα proteins were observed after 40 min of bicycling exercise at 70% of VO_2_max [15]. This disagreement is likely explainable by the fact that in Tantiwong et al. [15], the exercise was of moderate-intensity and not carried out until exhaustion or that the post-exercise muscle biopsy was slightly delayed since the subjects were moved from the cycle ergometer to a stretcher before taking the post-exercise muscle biopsy. Thus, it is critical to consider the timing of the post-exercise biopsies when interpreting skeletal muscle signalling responses, which should be reported in all studies.

Under resting conditions, NF-κB is bound to an inhibitor of κB proteins (IκBα, β, and ε), which keep NF-κB in the cytosol. Upon stimulation, IκBs are phosphorylated by IκB kinase (IKK), a trimeric enzyme constituted by two catalytic (IKKα and IKKβ) and one regulatory subunit (IKKγ). Their phosphorylation targets IκBs, and particularly IκBα, for proteasomal degradation, releasing its inhibitory action on NF-κB [45,46]. In agreement, IĸBα protein was reduced at the end of the incremental exercise and further reduced during ischaemia. However, in contrast with our hypothesis, these effects were not exacerbated when the exercise was carried out in severe acute hypoxia.

As a novelty, we have measured exercise-induced IĸBβ Thr^19^/Ser^23^ phosphorylation changes in human skeletal muscle. We have shown that IĸBβ Thr^19^/Ser^23^ phosphorylation increases remarkably in response to exercise to exhaustion, parallel with its upstream kinase IKKα/β, which was also phosphorylated and activated in response to exercise. The level of phosphorylation at Ser^176/180^ of IKKα/β and Thr^19^/Ser^23^ of IĸBβ were maintained in the leg recovering with ischaemia while it returned within 1 min after the cessation of exercise to pre-exercise levels in the leg recovering with free circulation. As expected, this response was paralleled by the changes in phospho-Thr^19^/Ser^23^ IĸBβ and phospho-Ser^536^ p65, which are known targets of IKKα [47].

Our results concur in part with two previous studies [16,17]. Firstly, in partial agreement with our results, Vella et al. [16] observed a reduction of total IĸBα and increase of phospho-Ser^536^ p65, but their first post-exercise biopsy was done 2 h after a single bout of resistance exercise. This finding by Vella et al. 16 could arise from the increased mitochondrial ROS production observed during the first hours after exercise [32]. Secondly, Petersen et al. [17] obtained muscle biopsies from eight well-trained men (VO_2_max 65 ml kg^−1^.min^−1^) after 45 min of exercise at 71% of VO_2_max and after exhaustion, since their subjects resumed exercise at 92% of VO_2_max until exhaustion immediately after the withdrawal of the 45 min biopsy. Opposed to Vella et al. [16], Petersen et al. did not see significant changes in p65 Ser^536^ phosphorylation immediately after exercise (in agreement with our findings), while IĸBα was reduced by 14% after 45 min of exercise and by 7% at exhaustion (the reduction observed at exhaustion was not statistically significant p = 0.06). Also in agreement with our results, Parker et al. [48] reported decreased IĸBα immediately after acute sprint interval exercise compared to less intense exercise modalities. Overall, the present findings and previous studies indicate that both the intensity of exercise and exhaustion favour the reduction of the inhibitor protein IκBα, facilitating the activation of NF-κB.

Exercise-related skeletal muscle changes in IĸBβ protein levels have not been previously reported, and the role that this NF-κB inhibitor may play in skeletal muscle physiology remains unknown. Here we have demonstrated a differential regulation of IĸBα and IĸBβ in human skeletal muscle: while the total amount of IĸBα is reduced with exercise and ischaemia, IĸBβ increases. A differential temporal regulation of IĸBs has been reported in cell cultures [49] and ageing hearts in mice [50]. In the present investigation, we have shown that both the phosphorylated and total form of IĸBβ protein are remarkably increased during high-intensity exercise, remaining elevated during ischaemic recovery. While phospho-Thr^19^/Ser^23^ IĸBβ returns to pre-exercise levels within 1 min of recovery with free circulation, the total amount of IĸBβ remained elevated 1 min after IE. This increase of IĸBβ with exercise may act as a negative feedback loop to impede excessive NF-κB activation. In addition, the increased expression of IĸBβ may contribute to the transcriptional specificity of NF-κB through the formation of the appropriate homo/heterodimers and the subsequent gene regulation [47,51]. In this regard, cell experiments have also shown that IĸBβ is a crucial mediator of the mitochondrial stress response [52] and is essential for the antioxidant response [51]. Moreover, overexpression of IĸBβ protects the liver against the ischaemia-reperfusion injury [53]. Thus, the linear association observed in the present investigation between IĸBβ and GR protein expression is compatible with a role of IĸBβ in the enhancement of skeletal muscle antioxidant capacity with regular intense exercise [[54], [55], [56], [57]].

### 4.2 The total amount of IKKα but not IKKβ is acutely increased in response to exercise with a similar response in normoxia and severe acute hypoxia

The effect of exercise on the protein levels of IKKα and IKKβ has not been previously studied in human skeletal muscle. Here we have observed differential regulation of these two catalytic subunits. In addition, we have detected a marked increase in the level of phosphorylation of IKKα/β Ser^176/180^, which was reverted within 1 min after the end of exercise. The latter may explain why no significant changes in IKKα/β Ser^176/180^ phosphorylation were observed immediately after a session of strength training [58]. In agreement with our results, increased IKKα/β Ser^176/180^ phosphorylation has been reported immediately after a single session of resistance, but not endurance exercise (2 h at 60% of VO_2_max) [59].

The activation of IKKs is necessary for the canonical stimulation of NF-κB signalling. In turn, IKKs phosphorylate p65 at Ser^536^ [60], which is necessary for its nuclear localization and protein stability and transcriptional activity [61,62]. The present investigation shows that post-exercise ischaemia promotes Ser^536^ p65 phosphorylation.

IKKs may be activated by autophosphorylation [47] and several upstream IKKs [47], among which only p38 and ERK1/2 have been mechanistically associated with contraction-induced NF-κB signalling in rodent muscle by inhibiting the two kinases pharmacologically [12]. In agreement with our results, it has been shown that ERK1/2 does not seem essential for NF-κB activation in cultured skeletal muscle cells [23]. Here, no increase in p38 MAPK phosphorylation was detected, in agreement with previous studies [63,64]. Nevertheless, increased p38 phosphorylation has been observed after repeated sprints [64,65] or prolonged continuous exercise [17,[65], [66], [67]], high-intensity repeated exercise [68], and intermittent exercise of moderate-intensity [69].

CaMKII has been shown to reduce IκB and activate NF-κB signalling [70]. More recently, direct phosphorylation of IKKβ by the delta isoform of CaMKII has been shown in cardiac fibroblasts [71]. In agreement, the present work shows a linear association between CaMKIIδ_D_ and the phosphorylated form of IKKα/β, supporting that CaMKII plays a similar role in skeletal muscle as reported in heart [70] and isolated cardiomyocytes [72].

### 4.3 Most of the NF-κB signalling induced by incremental exercise to exhaustion is quickly reverted to pre-exercise levels at exercise cessation unless metabolic recovery and re-oxygenation are prevented by the immediate application of ischaemia

All exercise-induced changes in NF-κB signalling, except the reduction in total IκBα, were reverted to pre-exercise values within 1 min from the end of exercise, showing that the deactivation of this signalling pathway is extremely fast, as previously shown in the heart [70]. In the present study, we used a novel experimental design to specifically determine whether muscle contractions are necessary to maintain NF-κB signalling. Immediately at the end of exercise, a pneumatic cuff was swiftly inflated at 300 mmHg to completely occlude the circulation in less than 2 s in one leg, while the contralateral leg recovered normally, i.e., with an intact circulation. At exhaustion, the muscle oxygenation was about 30% lower in hypoxia than normoxia [37]. Nonetheless, PCr and ATP were reduced, and lactate and H^+^ increased with similar responses at exhaustion in normoxia and hypoxia [37]. Since no significant differences were observed in muscle metabolites at exhaustion between normoxia and hypoxia, our results indicate that a lower oxygenation level *per se* does not elicit more NF-κB signalling.

The main differences between the contracting muscle at exhaustion and the muscle recovering under ischaemia were the interruption of Ca^2+^ transients due to the cessation of contractile activity, the absence of O_2_ during the ischaemic recovery and the lack of metabolic recovery. During the following 50s of ischaemia, lactate, H^+^, Pi and free creatine were increased, while no changes were observed in the concentration of ATP, which remained ∼20% below the pre-exercise values [37]. The Ca^2+^ transients elicited by muscle contractions are stopped at exhaustion in both legs. Since the increase of cytosolic Ca^2+^ has been shown to elicit NF-κB signalling in cells [8], it has been suggested that Ca^2+^-induced signalling could mediate the activation of NF-κB signalling in contracting muscles [12]. This is supported by the linear association between CaMKII δ_D_ phosphorylation and several critical molecules involved in NF-κB signalling in the present investigation.

Thus, the present findings indicate that the metabolites accumulated during the exercise and/or the lack of O_2_ may contribute to the maintenance of NF-κB signalling, likely by keeping CaMKII active. Interestingly, it has been reported that NF-κB contributes to stimulate glycolysis in C2C12 cells through activation of the glycolytic regulator hypoxia-inducible factor-1α (HIF-1α) [5]. Thus, the acute activation of NF-κB signalling during exercise and ischaemia may have contributed to upregulating the glycolytic energy production close to exhaustion and during the 60 s of ischaemia when the glycolysis provided more than 90% of the energy consumed [37].

In contrast, the recovery of ATP, PCr and the abundance of oxygen during recovery with open circulation may have facilitated CaMKII deactivation by the phosphatases, leading to downregulation of part of NF-κB signalling within seconds after the cessation of contractile activity.

### 4.4 RONS and NF-κB signalling during exercise and ischaemia in human skeletal muscle

Cell experiments have shown that NF-κB signalling may be stimulated by RONS [9,10,[73], [74], [75], [76]] and hypoxia [[21], [22], [23]]. RONS are produced in skeletal during exercise depending on exercise characteristics, the energy substrates oxidized, and fitness status [28,[77], [78], [79], [80]]. This process is facilitated by exercise conditions eliciting a robust stimulation of the glycolysis [77,81,82], as it occurs during exercise at high intensity and in hypoxia [77,78,81]. Using data from the same research project, we have reported a strong activation of the nuclear factor erythroid-derived 2-like 2 (Nrf2)/Kelch-like ECH-associated protein 1 (Keap1) signalling [33], which is activated by redox changes. The latter was associated with a remarkable increase of the antioxidant enzyme catalase, but not of superoxide dismutase 1 (Sod1) and Sod2 in the same biopsies studied here [33]. Thus, both Nrf2/Keap1 and NF-κB pathways are activated by exhausting exercise and post-exercise ischaemia, leading to an immediate increase of the antioxidant enzymes catalase (previously reported) [33] and GR, which is necessary to efficiently counteract superoxide and H_2_O_2_ [83]. In contrast, Trx1 content in skeletal muscle was decreased in the present investigation. This concurs with the secretion of Trx1 by C2C12 myotubes [84], and the observation of increased plasma levels of TRX1 60 min and 48 h following high-intensity exercise [85].

In cells, hypoxia (1% O_2_) inhibits prolyl hydroxylase-1 (PDH-1), which results in IKKβ activation, leading to IκBα phosphorylation and subsequent degradation [21]. Interestingly, hypoxia also facilitates the cellular response to cytokine-mediated stimulation of NF-κB [21]. Despite a remarkably lower femoral vein PO_2_ (and presumably in intracellular PO_2_) during exercise in severe acute hypoxia [37], no significant differences were observed between normoxia and hypoxia in any of the NF-κB signalling molecules assessed here.

In agreement with a RONS-dependent stimulation of NF-κB signalling during exercise, it has been reported that the administration before the exercise of allopurinol (a xanthine oxidase inhibitor) blunts NF-κB signalling by reducing RONS production in exercising rodents [24]. However, in humans, only one study has determined the effect of antioxidants (*N*- acetylcysteine infusion) administered before prolonged aerobic exercise on NF-κB signalling in skeletal muscle and no significant interactions were observed compared with placebo [17]. Thus, it remains to be determined whether antioxidants may prevent NF-κB signalling in exercising human skeletal muscles.

The present experiments demonstrate that ischaemia contributes to maintaining NF-κB signalling by impeding metabolite recovery and muscle re-oxygenation. Nevertheless, we have also observed that p65 + p50 and pSer^536^ NF-κB p65 were increased and IκBα reduced during ischaemia, indicating that post-exercise ischaemia stimulates NF-κB signalling further. The latter might have been facilitated by reducing cellular PO_2_ to anoxic levels. Thus, it seems that during incremental exercise to exhaustion in normoxia and severe acute hypoxia, NF-κB signalling is activated almost maximally and that further activation would require the application of post-exercise ischaemia to reduce muscle PO_2_ further or elicit a higher accumulation of metabolites. This finding implies that post-exercise ischaemia could be used to prolong the exercise-induced activation of NF-κB and the associated adaptive responses.

### 4.5 NF-κB activation is associated with the fast increase of glutathione reductase

NF-κB activation has been associated with the induction of antioxidant enzymes in several experimental models. For example, in TNF-α treated Ewing's sarcoma cells, NF-κB activation increased both thioredoxin and MnSOD levels [25]. Likewise, Glutathione S-transferase Pi, Metallothionein-3, NAD(P)H dehydrogenase [quinone]1, heme oxygenase-1 and glutathione peroxidase-1 have been shown to be induced by NF-κB [27]. Here, we show that the activation of NF-κB is positively associated with the protein expression levels of GR in human skeletal muscle. However, no similar association was observed for the other antioxidant enzymes tested.

In summary, this study shows a robust activation of NF-κB signalling with exercise to exhaustion which is not magnified by severe acute hypoxia and is maintained and further stimulated by ischaemia. These changes are quickly reverted at the end of exercise when the muscles recover with open circulation. Finally, our results indicate that a delay of just 1 min in obtaining the muscle biopsies can significantly impact the interpretation of exercise-induced NF-κB signalling in skeletal muscle.

## Disclosure summary

The authors have nothing to disclose.

## Appendix A Supplementary data

Multimedia component 1 Linear relationships between pThr^287^ CaMKII δ_D_ and protein markers. (A) pThr^287^ CaMKII δ_D_ and pSer^176/180^ IKK α/β, (B) pThr^287^ CaMKII δ_D_ and Total IKKα, (C) pThr^287^ CaMKII δ_D_ and NF-κB p105, (D) pThr^287^ CaMKII δ_D_ and Total IκB ß, (E) pThr^287^ CaMKII δ_D_ and pSer^536^ NF-κB p65, (F) pThr^287^ CaMKII δ_D_ and p65+p50 NF-κB, and (G) pThr^287^ CaMKII δ_D_ and GR. Linear relationships were assessed using a linear mixed model. The Likelihood Ratio Test for the random effects (LRT) was calculated and reported with the marginal (R^2^ marginal) and conditional (R^2^ conditional) r-squared values (n = 73-75). Multimedia component 1

Multimedia component 2 Linear relationships between protein markers. (A) NF-κB p105 and NF-κB p50, and (B) Total IκBß and GR. Linear relationships were assessed using a linear mixed model. The Likelihood Ratio Test for the random effects (LRT) was computed and reported with the marginal (R^2^ marginal) and conditional (R^2^ conditional) r-squared values (n = 73-75). Multimedia component 2
