# Supplementary figures and images for: Fast regulation of the NF-κB signalling pathway in human skeletal muscle revealed by high-intensity exercise and ischaemia at exhaustion: Role of oxygenation and metabolite accumulation
Source: Redox Biol. 2022 Jul 8;55:102398. doi: 10.1016/j.redox.2022.102398 (PMC9287614; doi:10.1016/j.redox.2022.102398)

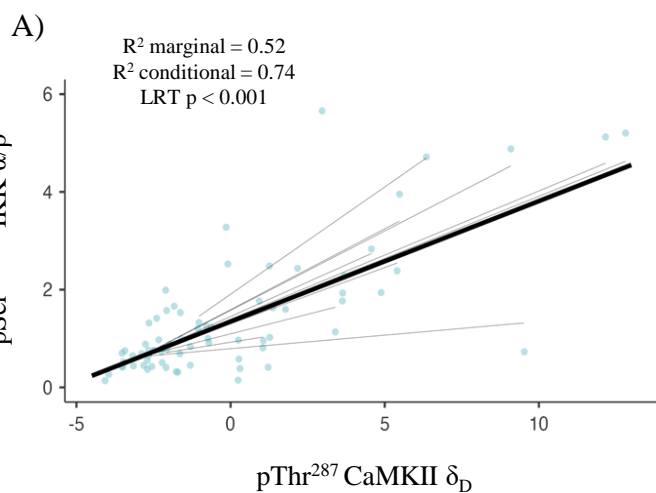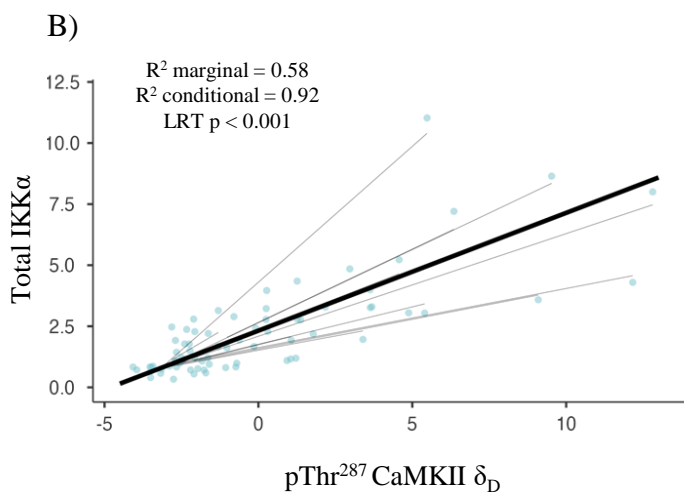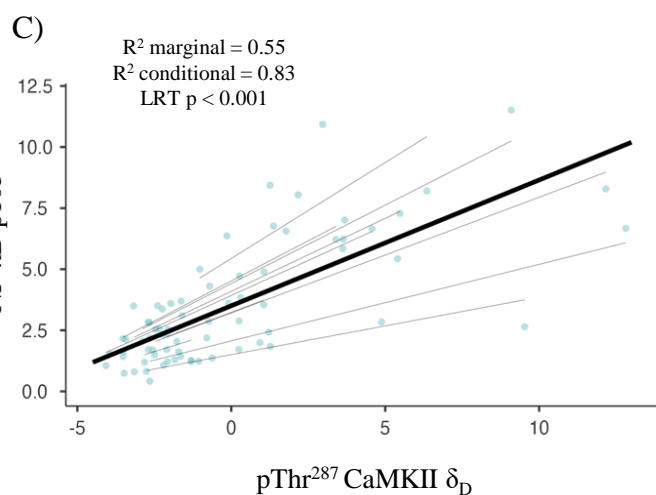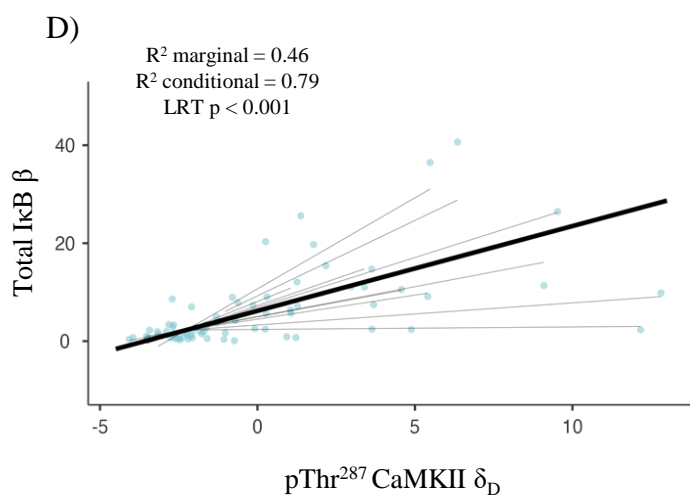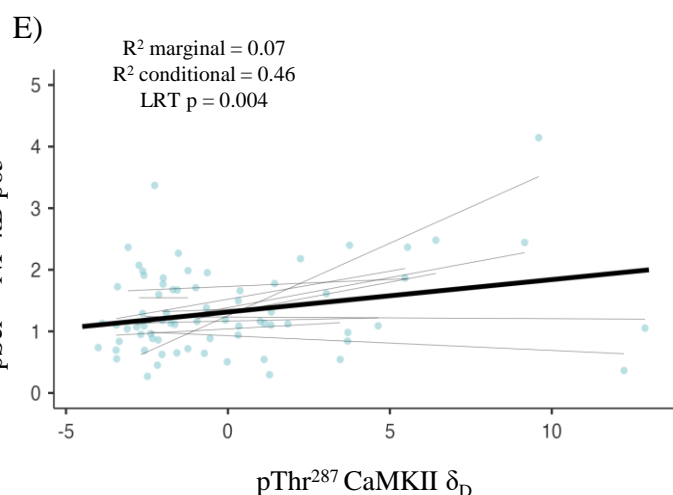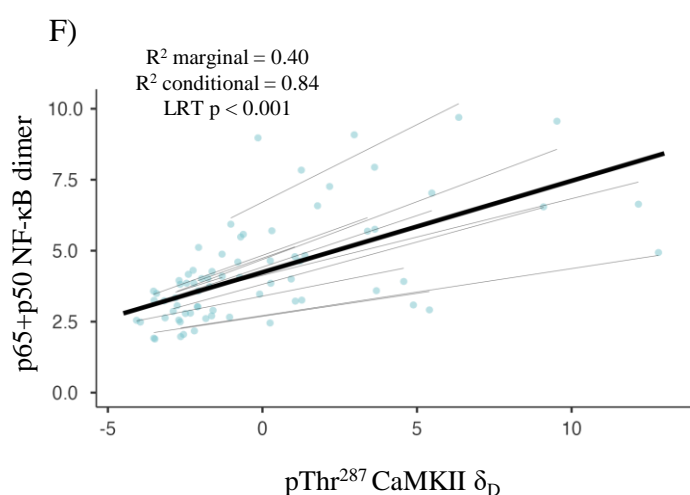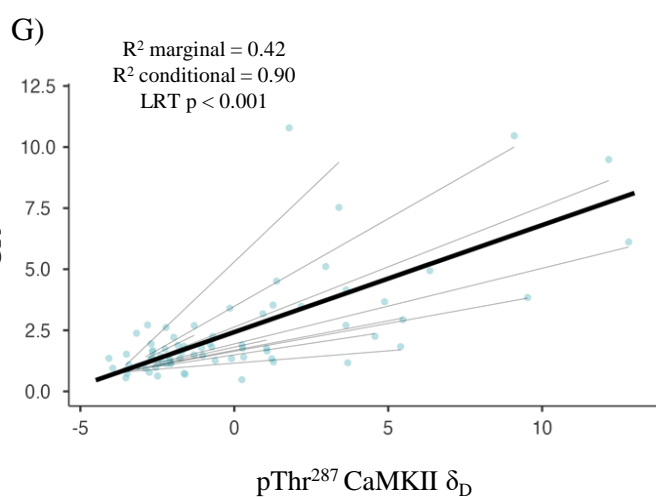

Supplement: Multimedia component 1 — Linear relationships between pThr287 CaMKII δD and protein markers. (A) pThr287 CaMKII δD and pSer176/180 IKK α/β, (B) pThr287 CaMKII δD and Total IKKα, (C) pThr287 CaMKII δD and NF-κB p105, (D) pThr287 CaMKII δD and Total IκB ß, (E) pThr287 CaMKII δD and pSer536 NF-κB p65, (F) pThr287 CaMKII δD and p65+p50 NF-κB, and (G) pThr287 CaMKII δD and GR. Linear relationships were assessed using a linear mixed model. The Likelihood Ratio Test for the random effects (LRT) was calculated and reported with the marginal (R2 marginal) and conditional (R2 conditional) r-squared values (n = 73-75). [file mmc1.pdf]

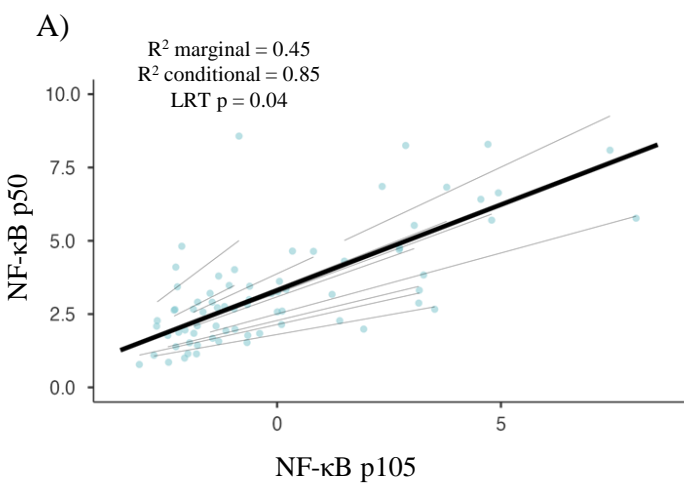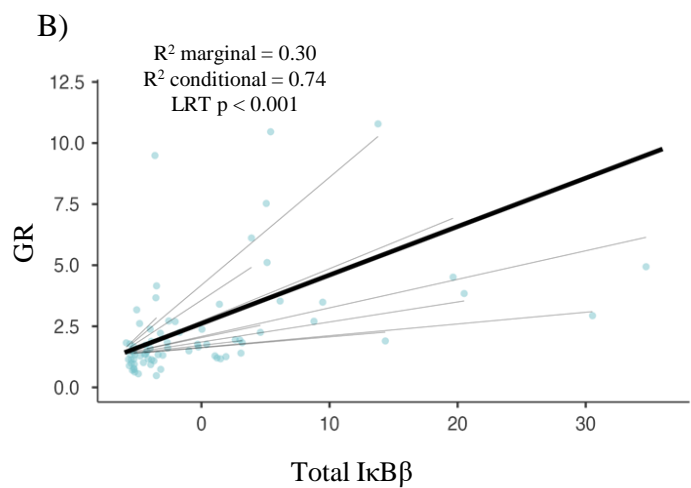

Supplement: Multimedia component 2 — Linear relationships between protein markers. (A) NF-κB p105 and NF-κB p50, and (B) Total IκBß and GR. Linear relationships were assessed using a linear mixed model. The Likelihood Ratio Test for the random effects (LRT) was computed and reported with the marginal (R2 marginal) and conditional (R2 conditional) r-squared values (n = 73-75). [file mmc2.pdf]
