# Supplementary material for: Fast regulation of the NF-κB signalling pathway in human skeletal muscle revealed by high-intensity exercise and ischaemia at exhaustion: Role of oxygenation and metabolite accumulation
Source: Redox Biol. 2022 Jul 8;55:102398. doi: 10.1016/j.redox.2022.102398 (PMC9287614; doi:10.1016/j.redox.2022.102398)
Supplement: Multimedia component 3 [file mmc3.docx]

**Supplementary Table 1: Detailed description of Western blotting antibodies and procedures.**

| **Antibody** |  | **Manufacturer company** |  | **Catalog number** |  |  | **Gel %** |  | **Protein amount loaded**  **(μg)** |  | **Blotting transfer time (min)** |  | **Blocking reagent** |  | **Primary antibody concentration** |  | **Secondary**  **antibody concentration** |
| --- | --- | --- | --- | --- | --- | --- | --- | --- | --- | --- | --- | --- | --- | --- | --- | --- | --- |
| Thr^287^  CaMKII |  | Cell Signaling |  | 12716 |  |  | 10 |  | 20 |  | 90 |  | BSA 4% |  | 1:2000 |  | 1:5000 |
| Total CaMKII |  | Cell Signaling |  | 4436 |  |  | 10 |  | 12.5 |  | 90 |  | BSA 4% |  | 1:1000 |  | 1:5000 |
| pThr^180^/Tyr^182^ p38 MAPK |  | Cell Signaling |  | 9211 |  |  | 7.5-10 |  | 10 |  | 90 |  | Blotto 5% |  | 1:3000 |  | 1:20.000 |
| pThr^202^/Tyr^204^ ERK 1/2 |  | Cell Signaling |  | 9106 |  |  | 10 |  | 12.5 |  | 90 |  | BSA 4% |  | 1:1000 |  | 1:20.000 |
| Total ERK 1/2 |  | Cell Signaling |  | 9102 |  |  | 10 |  | 10 |  | 90 |  | Blotto 1% |  | 1:1000 |  | 1:20.000 |
| NFκB p105 |  | Cell Signaling |  | 13586 |  |  | 4-20 |  | 30 |  | 90 |  | Blotto 5% |  | 1:2000 |  | 1:5.000 |
| NFκB p50 |  | Cell Signaling |  | 13586 |  |  | 4-20 |  | 30 |  | 90 |  | Blotto 5% |  | 1:2000 |  | 1:5.000 |
| Total NFκB p65 |  | Cell Signaling |  | 3034 |  |  | 10 |  | 22.5 |  | 90 |  | BSA 4% |  | 1:2000 |  | 1:10.000 |
| pSer^536^ NFκB p65 |  | Cell Signaling |  | 3033 |  |  | 4-20 |  | 15 |  | 90 |  | BSA 4% |  | 1:5000 |  | 1:5.000 |
| pSer^32/36^ IκBα |  | Cell Signaling |  | 9246 |  |  | 10 |  | 10 |  | 90 |  | BSA 4% |  | 1:2000 |  | 1:10.000 |
| Total IκBα |  | Cell Signaling |  | 9242 |  |  | 4-20 |  | 7.5 |  | 90 |  | BSA 4% |  | 1:2000 |  | 1:10.000 |
| pThr^19^/Ser^23^ IκBß |  | Cell Signaling |  | 4921 |  |  | 7.5-10 |  | 10 |  | 90 |  | BSA 4% |  | 1:1000 |  | 1:10.000 |
| Total IκBß |  | Abcam |  | ab109509 |  |  | 10 |  | 10 |  | 90 |  | Blotto 5% |  | 1:3000 |  | 1:20.000 |
| pSer^176/180^ IKKα/ß |  | Cell Signaling |  | 2697 |  |  | 4-20 |  | 12.5 |  | 90 |  | BSA 4% |  | 1:3000 |  | 1:20.000 |
| Total IKKß |  | Cell Signaling |  | 2370 |  |  | 4-20 |  | 12.5 |  | 90 |  | BSA 4% |  | 1:3000 |  | 1:10.000 |
| Total IKKα |  | Cell Signaling |  | 2682 |  |  | 4-20 |  | 15 |  | 90 |  | BSA 4% |  | 1:3000 |  | 1:20.000 |
| GSR |  | ProteinTech |  | 18257-1-AP |  |  | 10 |  | 5 |  | 90 |  | Blotto 2.5% |  | 1:2000 |  | 1:10.000 |
| TXR1 |  | Cell Signaling |  | 2429 |  |  | 15 |  | 12.5 |  | 90 |  | Blotto 2.5% |  | 1:2000 |  | 1:10.000 |
| GPX1 |  | Abcam |  | ab108429 |  |  | 15 |  | 12.5 |  | 90 |  | Blotto 2.5% |  | 1:2000 |  | 1:10.000 |
| TXNRD1 |  | ProteinTech |  | 11117-1-AP |  |  | 10 |  | 5 |  | 90 |  | Blotto 2.5% |  | 1:2000 |  | 1:10.000 |
